# Relevant Aspects of *Clostridium estertheticum* as a Specific Spoilage Organism of Vacuum-Packed Meat

**DOI:** 10.3390/microorganisms7050142

**Published:** 2019-05-20

**Authors:** Joseph Wambui, Roger Stephan

**Affiliations:** Institute for Food Safety and Hygiene, Vetsuisse Faculty, University of Zurich, Winterthurerstrasse 272, 8057 Zurich, Switzerland; stephanr@fsafety.uzh.ch

**Keywords:** *Clostridium estertheticum*, blown pack spoilage, vacuum-packed meat, meat spoilage

## Abstract

*Clostridium estertheticum* is a psychrotolerant, gram-positive, motile, anaerobic, spore-forming, rod-shaped bacteria that causes blown pack spoilage (BPS). Spoilage occurs in vacuum-packed meat without temperature abuse. Having been reported in the last 30 years in several countries, BPS by *Cl. estertheticum* is a major issue around the world and presents a huge economic impact on the meat industry. Despite being an important spoilage microorganism, studies on *Cl. estertheticum* are challenged by numerous aspects. These include, lack or poor growth in laboratory media, long culturing periods, and unpredictable isolation on the media. These factors hamper the detection of *Cl. estertheticum* before occurrence of BPS, which further undermines efforts to prevent the occurrence of BPS. Nevertheless, considerable developments have taken place with regard to culture-independent methods. Although information on *Cl. estertheticum* is available, it is limited and remains highly fragmented. Therefore, this review collates the available information and discusses relevant aspects of *Cl. estertheticum* as a specific spoilage organism of BPS in vacuum-packed meat.

## 1. Introduction

Meat is a highly perishable food commodity hence prone to microbial or chemical spoilage. Meat spoilage refers to alteration of color and production of off-odors, slime, and exudates that lead to unacceptable sensorial and organoleptic properties [1]. Spoilage of meat can have a significant effect on the global food supply. In Europe and Northern America, approximately 21% of food losses are from meat and meat products [2]. On the other hand, meat spoilage accounts for up to 40% of the production losses incurred by the meat processors and retailers [3]. These factors result in huge financial losses to the meat industry and are also a big issue in view of sustainability.

Meat spoilage results from a combination of microbial and chemical activities. Both are considered important, although microbial activities are a major cause of spoilage especially for raw meats [4,5]. Meat is generally considered sterile before slaughter, but the environment in which slaughter processes take place are not sterile, and therefore a degree of microbial contamination can occur [6], resulting in microbial spoilage. Post-slaughter microbial quality of meat is primarily determined by meat type, processing, distribution, and storage conditions [7]. Contaminated slaughter equipment, personnel, and environmental factors, such as water, air, and soil, can cross contaminate meat with spoilage-related bacteria species [8,9]. Upon storage, various intrinsic and extrinsic factors influence the process of microbial meat spoilage. These include oxygen demand, pH, temperature, and competing organisms [10]. The diversity of these ecophysiological factors affect the microbial growth dynamics, including succession of microorganisms and the microbiota composition and ultimately the type and rate of meat spoilage.

The ecosystem of meat offers easily available substrates among them glycogen and amino acids. The nutrients provide an enabling environment for diverse microbial growth and metabolism that results in spoilage [11]. Despite this, only a fraction of the initial microbial population on meat, which are referred to as ‘specific spoilage organisms’ (SSO) can develop during storage and cause spoilage [12,13]. In meat, the SSO metabolize available substrates, with subsequent changes in meat texture and production of volatile organic compounds responsible for off-odors [14]. SSO can also cause accumulation of purge especially in vacuum-packing [15].

Vacuum-packing is used by meat processors to control meat spoilage during storage. Its ability to prevent the growth of some food-borne pathogens and spoilage bacteria commonly present on meat makes it a widely used meat packing method [16]. In the vacuum-packing system, an anaerobic environment is created by removing oxygen followed by immediate sealing [17]. It is generally recognized that meat packing systems are important extrinsic factors that determines SSO in stored meats [18]. Effectively, exhaustion of oxygen alters the gaseous composition within the packs creating hurdles to the aerobic bacteria while enabling the growth of facultative and strict anaerobes [19].

In addition to creating anaerobic conditions, vacuum-packing maintains the pH of meat between 5.0 to 6.0 under long storage [20]. Lactic acid content in meat, which results from metabolism of glycogen by some anaerobic SSO [21], may also affect the growth of other SSO in vacuum packed meat by not only lowering the pH of meat but also favoring microorganisms that can metabolize it [22]. Cold storage of vacuum-packed meat further selects for psychrophilic or psychrotolerant SSO [12]. The main SSO in vacuum-packed meat include *Streptococcus* spp., *Brochothrix* spp., *Psychrobacter* spp., and *Acinetobacter* spp. [8,23,24]. A range of psychrotolerant and psychrophilic Clostridia have also been identified as SSO of chilled, vacuum-packed red meat [25].

Among the Clostridia, *Clostridium estertheticum*, *Cl. algidicarnis*, *Cl. frigidicarnis*, *Cl. gasigenes*, *Cl. Frigoris,* and *Cl. bowmanii* are particularly involved in spoilage of chilled vacuum-packed meat and meat products [26,27]. Spoilage of vacuum-packed meat by *Cl. algidicarnis*, *Cl. frigoris*, *Cl. bowmanii*, and *Cl. frigidicarmis* occurs without gas production while spoilage by *Cl. estertheticum* and *Cl. gasigenes* is characterized by gas production [25]. The spoilage by *Cl. estertheticum* and *Cl. gasigenes* is commonly referred to as ‘blown pack spoilage’ (BPS), and *Cl. estertheticum* is regarded as the main cause of BPS [28], making it the main SSO of BPS. 

There are two recognized subspecies of *Cl. estertheticum*, *Cl. estertheticum* subp. *estertheticum* and *Cl. estertheticum* subp. *laramiense* [29,30], both of which are linked to BPS [31]. The first reports of BPS by *Cl. estertheticum* were made in 1989 of vacuum-packed raw beef in the UK and USA [32,33]. Later, BPS was reported in New Zealand [34] and Ireland [35], making it a global phenomenon with an impact on the meat industry. The present study reviews the characteristics of *Cl. estertheticum* as an SSO of BPS in refrigerated vacuum-packed meat.

## 2. Taxonomic Classification of *Clostridium estertheticum*

The *Clostridium* genus is a large, diverse group consisting of Gram-positive, spore-forming, obligate anaerobic firmicutes [36]. The genus was created in 1880 with the type species *Cl. butyricum* [37]. Currently, the genus consists of over 230 recognized species and subspecies [38]. This is attributed to the wide range of phenotypes displayed by the different species within the genus, which include synthesis of quinone and cytochromes, varying glycine and cysteine (GC) content, and wide growth temperature and acidity ranges [39]. The application of molecular methods, which include DNA–rRNA pairing and 16S rRNA cataloguing studies, have revealed the phylogenetic diversity and resulted in reclassification of some species within the genus [39,40]. Based on the phylogenetic analysis, the genus is currently divided into Cluster I and Cluster II with less than 80 species, among them *Cl. estertheticum*, falling into the Cluster I and are referred to as *Clostridium sensu stricto* [29,30].

Despite having been first identified and reported in the same year, different names were assigned to the species *Cl. estertheticum* [32,33], possibly because they were isolated in different countries. The organism identified in spoilt vacuum-packed meat in UK, was named *Cl. estertheticum* due to its ability to form esters [41]. On the other hand, the organism identified in spoilt vacuum-packed meat in the USA was named *Cl. laramie* in reference to the City of Laramie, Wyoming [42]. Collins [41] characterized the organism through 16S ribosomal RNA (rRNA) while Kalchayanand et al. [42] characterized it through phenotypic tests and its GC content. The taxonomy of the two species was later resolved when DNA–DNA hybridization experiments revealed that they shared 79% DNA–DNA similarity and 16s RNA analysis revealed they formed a tight cluster, indicating a close relationship at the species level [43]. Therefore, Spring [43] proposed that the two species be merged into one species, named *Cl. estertheticum*, which was divided into two subspecies, *Cl. estertheticum* subsp. *estertheticum* and *Cl. estertheticum* subsp. *laramiense*.

Sequencing of *Cl. estertheticum* DSM 8809 strain, revealed that it harbors a marginally higher GC content than *Cl. botulinum* and *Cl. perfringens* [44]. On the other hand, *Cl. estertheticum* subsp. *estertheticum* and *Cl. estertheticum* subsp. *laramiense* do not cluster together in a phylogenetic tree despite their 16s RNA sequence being similar [30,43]. In particular, *Cl. estertheticum* subsp. *laramiense* clusters with *Cl. frigioris,* while *Cl. estertheticum* subsp. *estertheticum* clusters with *Cl. lacusfryxellense* in the range of 98.7–99.6% [30,43]. These differences have raised questions of classifying the two subspecies as a single species under the current classification system [45]. With respect to phenotypic characteristics, the two subspecies were initially reported to have differences in hemolytic activity, growth conditions, spore position and fermentation products [41,43]. These differences formed the basis for characterizing the two subspecies of *Cl. estertheticum*. It was later reported that the two organisms did not display these differences [45]. This exemplifies the challenges faced when using phenotypic traits within the *Clostridium* genus for taxonomic classification.

## 3. Isolation and Conventional Culturing of *Clostridium estertheticum*

Even though *Cl. estertheticum* was first reported in 1989, its isolation was hampered by its failure to grow in laboratory media available at the time, which included thioglycollate agar, brain heart infusion agar, lactose egg-yolk-milk agar, and trypticase peptone glucose yeast extract agar [32,33]. Similar results were reported 20 years later by Byrne et al. [35], whereby their efforts to isolate *Cl. estertheticum* from vacuum-packed purge in BPS samples using Colombia blood agar, tryptose sulphite cycloserine, Shahidi-Ferguson perfringens agar, reinforced clostridial agar, and brain-heart infusion agar resulted only in growth of other facultative anaerobic non-spore-forming bacteria other than *Cl. estertheticum*. Nevertheless, pure cultures of *Cl. estertheticum* could initially be grown on Reinforced Clostridium Medium (RCM) [41]. Later, Broda et al. [46] developed a pre-reduced RCM-based protocol that involved treatment of samples with ethanol and heat to recover Clostridia spores after inactivating other microorganisms and applied it to isolate *Clostridium* spp. from vacuum-packed purge samples. This method was used by Boerema et al. [47] to isolate *Cl. estertheticum* from slaughterhouse processing environments, which could then be identified on colony-based morphologies on agar supplemented with blood. The method was later compared against a method that included an enrichment step using pre-reduced peptone yeast extract glucose starch (PYGS) medium (Table 1) in isolation of *Cl. estertheticum* from abattoir samples and it was shown that the enrichment step enhanced the successful isolation of the organism [48].

While unsuccessful culturing of *Cl. estertheticum* has been attributed to the production of butanol within isolation media that eventually kills the organism’s cells [49], there are currently no dedicated media to overcome this challenge. Furthermore, there are no differential media for its identification due to variable phenotypic characteristics between the two subspecies [41,43,45,48] and among psychrophilic Clostridia [50]. For these reasons, *Cl. estertheticum* is not detectable through most culturing methods that are presently available [51]. Therefore, culture-based methods for *Cl. estertheticum* have, over the years, been based on non-specific media including PYGS medium and Columbia blood agar (CBA) supplemented with 5% defibrinated horse blood [52]. On CBA, *Cl. estertheticum* forms colonies that are round with often coarsely granulated margins, smooth, slightly raised, cream-white to greyish and semitransparent to opaque and can either be or not be β-hemolytic [48]. The current conventional based culture processes are time consuming because *Cl. estertheticum*’s optimal temperature for growth is low, hence production of a workable culture is slow, often taking up to three months [44]. Even with a working culture, lack of a comprehensive list of bacterial species in currently available commercial kits, which allow for phenotypic differentiation of the species, may further hamper the correct identification of *Cl. estertheticum* [53].

## 4. Molecular and Non-Molecular Based Identification of *Clostridium estertheticum*

Despite the different challenges of culture-dependent method, molecular methods, such as polymerase chain reaction (PCR), have proven reliable for detection of *Cl. estertheticum*. The success of some the methods is dependent on an enrichment step before molecular analysis [28,54]. Collins [41] described the first molecular based detection method for *Cl. estertheticum*, which was based on the 16S rRNA gene that allowed the differentiation of *Cl. estertheticum* from *Cl. acetobutylicum*, *Cl. aurantibutyricum* and *Cl. tetanomorphum*. Thereafter, two ribosomal DNA (rDNA) based methods were developed to detect *Cl. estertheticum* in broth, meat or meat purge [55]. Broda et al. [34] developed a 16S rDNA gene-based restriction fragment length polymorphism (RFLP) analysis for *Cl. estertheticum* differentiation from *Cl. botulinum*, *Cl. algidicarnis*, *Cl. putrefaciens*, *Cl. Vincentii,* and *Cl. fimetarium*. In an additional study, Broda et al. [56] used an 16S-23S rDNA internal transcribed spacer analysis to detect *Cl. estertheticum* in the meat processing environment. Broda et al. [57] then described the first set of primers, 16SEF and 16SER, which allowed the differentiation of *Cl. estertheticum* from other closely related Clostridia and microorganisms found in meat without RFLP analysis. The protocol was also suitable for the detection of *Cl. estertheticum* in commercial blown packs with a detection limit of 100 *Cl. estertheticum* cells per gram. This method was thereafter used and validated in different further studies [47,58,59].

In an effort to reduce detection time for *Cl. estertheticum*, Brightwell and Clemens [28] developed and validated a real-time PCR (RT-PCR) assay that was applicable in a variety of matrices including soil, hides, feces and meat (Table 2). Bonke et al. [50] compared this RT-PCR assay to the conventional PCR method described by Broda et al. [57], and showed that the RT-PCR assay was more sensitive. Based on the RT-PCR developed by Brightwell and Clemens [28], Reid et al. [60] developed and validated a further RT-PCR method for the simultaneous detection of low concentrations *Cl. estertheticum*, *Cl. gasigenes* and *Cl. ruminantium* in meat juice and wet or dry swab samples. The RT-PCR could detect five spores per milliliter without the need of an enrichment step, hence also speeding up the time taken to identify *Cl. estertheticum* considerably. Recently, Dorn-In et al. [25] developed a multiplex quantitative-PCR (q-PCR) (Table 2) for the detection of *Cl. estertheticum* as well as *Cl. frigoriphilum*, *Cl. bowmanii* or *Cl. tagluense* and showed that the q-PCR could be applied directly with DNA extracts of meat juice from BPS samples.

*Cl. estertheticum* subsp. *estertheticum,* and *Cl. estertheticum* subsp. *laramiense* could be differentiated by *Sma*I digestion of their DNA followed by pulsed-field gel electrophoresis (PFGE) analysis [45]. The PFGE analysis could differentiate between the two subspecies because of their distinctly different PFGE patterns, with a Dice similarity coefficient of 90%. Most recently, Amplified rDNA Restriction Analysis (ARDRA), which is a modified RFLP method, was used to differentiate *Cl. estertheticum* from other spoilage-associated species of Clostridia as well as other pyschrotolerant *Clostridium* species associated with meat production [61]. Most recently, Matrix Assisted Laser Desorption Ionization-Time of Flight Mass Spectrometry (MALDI-TOF MS) was used to identify *Cl. estertheticum* isolated from sheep and cattle carcasses at different slaughtering stages, and the results verified using 16S rDNA gene sequencing [53].

## 5. Growth and Metabolism of *Clostridium estertheticum*

*Cl. estertheticum* is an obligate anaerobe bacterium, hence it is sensitive to oxygen when in vegetative state [62]. Being a psychrotolerant Clostridia, *Cl. estertheticum* grows in a temperature range between −2 and 22 °C [63,64], and does not grow at 25 °C or above [41]. The optimum growth temperature ranges between 6–15 °C [43,45]. The type of substrate may also influence the growth temperatures given that *Cl. estertheticum* grew in meat juice at 20 °C but not in PYGS broth [63]. *Cl. estertheticum* grows at pH values in the range of 5.5 to 7.5 with maximum growth occurring between 5.8 and 6.8 [63]. In a growth assay in meat juice, *Cl. estertheticum* utilized both glucose and glycogen for growth, but exhaustion of glucose resulted in cessation of growth with a simultaneous utilization of lactate and production of CO_2_ and H_2_, but without growth probably due to low levels of acetate [65]. *Cl. estertheticum* produced butyrate, acetate, and formate from glucose and 1-butanol, ethanol, butyrate, and formate from lactate [45]. *Cl. estertheticum* also utilized amino acids that do not contain Sulphur for growth hence do not produce hydrogen sulfide [45]. Meat with a high pH and glucose concentration is the most conducive food matrix for the growth of *Cl. estertheticum* [66].

## 6. *Clostridium estertheticum* as a Causative Agent of Blown Pack Spoilage

It has been previously reported that BPS during refrigeration can also be caused by gas-producing *Enterobacteriaceae*, including *Hafnia* spp., *Enterobacter* spp., *Serratia* spp., *Rahnella* spp., and *Ewingella* spp. [67]. Nevertheless, these species do not grow below 4 °C [68]. Even though different SSO, including the *Enterobacteriaceae* and lactic acid bacteria, can cause BPS at refrigeration temperatures of 4 °C to 15 °C [69], the spoilage below 2 °C would be a major characteristic for BPS by *Cl. estertheticum* [46].

A four-year survey of BPS in Ireland found that the prevalence of *Cl. estertheticum* was higher than that of other Clostridia [70]. In another study, beef and lamb samples from Europe, North and South America and Oceania were investigated and it was also found that *Cl. estertheticum* was the most prevalent Clostridia [50]. A comparison among 11 *Clostridium* species found that all species were able to grow on vacuum-packed meat but only *Cl. estertheticum* and *Cl. frigioris* caused BPS [71]. Similar results were obtained by Silva et al. [72]. These reports emphasize that *Cl. estertheticum* is by far the most common psychrotolerant Clostridia associated with BPS of vacuum-packed raw meats, hence an SSO of refrigerated vacuum-packed meat below 2 °C.

Typically, a pack suffering from BPS caused by *Cl. estertheticum* contains copious quantities of drip and gas, whereby the latter leads to gross pack distension [64]. Upon opening of dissented packs, BPS may be characterized by either a highly unpleasant odor followed by a very strong fruity and dairy odors [33,63]. The three odors cannot be used conclusively to characterize BPS by *Cl. estertheticum* given that, despite its inability to produce hydrogen sulfide [45], an unpleasant odor was perceived from a naturally spoilt pack [32], but absent in another study [63]. Spoiled meat in BPS packs is nonetheless discolored and excessively tender [33].

BPS is not regarded as a safety hazard, but meat spoiled in this way has no commercial value and causes significant financial losses to the meat industry [70]. Losses that are specifically caused by *Cl. estertheticum* are likely to occur in the summer, which is the season with the highest prevalence of *Cl. estertheticum* [70,73]. Although the safety risks associated with *Cl. estertheticum* are regarded to be low, genome analysis revealed that *Cl. estertheticum* harbors multiple genes potentially related to antibiotic, biocide and metal resistance, along with several predicted virulence factor genes [44].

## 7. Factors Affecting Blown Pack Spoilage by *Clostridium estertheticum*

Being anaerobic, BPS by *Cl. estertheticum* is primarily dependent on the presence of spores on meat prior to packing and their ability to germinate within vacuum packs. As low as one spore of *Cl. estertheticum* is sufficient to cause BPS [64]. It was suggested that 100 spores per cm^2^ of *Cl. estertheticum* is a critical number for vacuum-packed meat [74]. The rate of spore germination, hence the ratio of spores and vegetative cells during the ongoing process of BPS, can also influence the occurrence of BPS [75]. In particular, both neutral pH and lactate increase the rate of spore germination [62]. On the other hand, the maximum numbers of *Cl. estertheticum* in the spoilage flora of vacuum-packed meat was shown to depend on the amount of glucose available for its growth [65]. Therefore, the amount of glucose in meat after slaughter is a key factor because it is the first substrate preferably utilized by most bacteria growing in raw meat during refrigerated storage [14]. Moreover, the ability of *Cl. estertheticum* to compete with microflora that utilize glucose, such as *Leuconostoc mesenteroides* in vacuum-packed meat can influence the occurrence of BPS [76].

Storage temperature has an influence on the occurrence of BPS. In order to minimize meat spoilage by anaerobic bacteria, a storage temperature of −1.5 °C has been recommended, and in vacuum-packed meat, temperature above 0 °C is considered abusive [77]. The shelf life for vacuum-packed meat stored at −1.5 °C ranges between 60 to 70 days [78]. Unfortunately, BPS by *Cl. estertheticum* can occur in the absence of temperature abuse or packaging failure [59]. As low as 10 spores per cm^2^ of *Cl. estertheticum* can reduce the shelf life of vacuum-packed meat to 44 days at −1.5 °C [64]. At 2 °C and 15 °C, packs inoculated with *Cl. estertheticum* presented the first signs of BPS after 15 and 4 days of storage, respectively [72]. While storage of meat at −1.5 °C evidently slows the rate of BPS, the time it takes for the meat to reach −1.5 °C after chilling had no effect on the occurrence of BPS [75]. Another temperature related factor is the application of heat shrink. This involves dipping vacuum packs of meats in water at 85–90 °C, for 2–3 s immediately after pack sealing to improve the pack integrity [79]. This practice has been shown to accelerate the onset of BPS by *Cl. estertheticum* whereby gas was produced in heat-treated packs much earlier than pack without heat treatment [80].

Occurrence of BPS can also be influenced by post-slaughter deboning of carcasses. Deboning can either be hot-boning, which involves deboning before chilling or cold-boning, which involves deboning after chilling [81]. Hot-boning has numerous economic and technological benefits that include less chill and drip loss, cooler space, electricity, and capital investment and results in high quality meat in terms of high pH, water-holding capacity, and emulsifying capacity [82,83]. A comparison of the two deboning techniques showed that hot-boning resulted in earlier detection of BPS caused by *Cl. estertheticum* than in cold-boning [60].

## 8. Intervention and Inactivation Strategies to Reduce Blown Pack Spoilage by *Clostridium estertheticum*

The process of BPS occurs suddenly and is only detectable when packs are dissented indicating that contamination with *Cl. estertheticum* occurred pre-packing. *Cl. estertheticum* spores have been found on carcasses including primal cuts [53,70]. However, meat model experiments show that meat without artificially inoculated spores of *Cl. estertheticum* did not show signs of BPS during the entire duration of the trial 12–15 weeks [51,58,79]. Evidently, contamination of meat with *Cl. estertheticum* spores before packaging plays an important role in occurrence of BPS. These spores are usually transferred onto dressed carcasses during slaughter and processing [47,56]. The control of *Cl. estertheticum* must therefore be eliminated from the meat-processing environment, including stockyard pens, slaughter floor and soil, by means of extensive cleaning and sanitizing [59]. BPS in a meat plant can also be controlled by developing technologies to remove, kill, or inactivate spores of *Cl. estertheticum* present on dressed carcasses [58]. Given that *Cl. estertheticum* spores are resistant to heat, freezing, many chemicals and harsh environments [49], highly innovative and effective strategies would be required.

A comparison of three inactivation methods for *Cl. estertheticum* spores found that use of heat alone or ultrasound followed by heat treatment resulted in incomplete spore inactivation while peroxyacetic acid (POAA) sanitizer used with or without heat resulted in at least 4 log CFU per milliliter *Cl. estertheticum* spores at ambient temperature [49]. A similar effect of POAA on *Cl. estertheticum* was observed whereby BPS was delayed with a higher impact observed at −1.5 °C than 0 °C and 2 °C [58]. A POAA spray treatment on stainless steel coupons for 1 min resulted in a 2.3 log CFU per coupon reduction of *Cl. estertheticum* spores [84]. In the same experiment, a hydrogen peroxide vapor treatment for 150 min effectively inactivated *Cl. estertheticum* spores on the stainless-steel coupons [84]. On other hand, a hydrogen peroxide (15%) gel treatment for ten minutes on raw and pre-washed fleece resulted in a significant reduction of *Cl. estertheticum* spores [84]. Incubation of spores in meat juice at 80 °C for 10 min resulted in a 0.7 log CFU reduction in spore numbers, due to a likely loss of heat resistance of germinating spores [62].

In an in vitro experiment, *Cl. estertheticum* was inhibited by cultures of *Lactococcus lactis*, *Lactobacillus sakei*, *Lactococcus garvieae* and *Leuconostoc carnosum* but not by their cell-free fractions [73]. Similar results were reproduced in a meat model system, whereby BPS was delayed in meat inoculation with both *Cl. estertheticum* and *L. lactis* compared to inoculation with *Cl. estertheticum* only [51]. In an experiment to compare commercially available antimicrobials, Auranta FV (AFV; composed of bioflavonoids, citric, malic, lactic, and caprylic acids), Inbac-MDA (IMDA; composed of sodium diacetate, malic acid, mono and diglycerides of fatty acids, salt and excipients), and sodium octanoate (SO) incorporated in active packaging systems, found that AFV and SO prevented BPS within 42 days [85]. Times and temperature combinations of 240 s at 100 °C were also shown to inactivate *Cl. estertheticum* spores [49].

While the application of heat shrink after vacuum-packing may activate spores, the step is an invaluable processing step in meat processing. Initially, Bell et al. [80] recommended the application of other techniques besides heat shrink to achieve similar benefits. Later, several authors studied the practice in relation to BPS and recommended best practices for its application. Moschonas et al. [79] recommended to perform it at 50 °C for 15 s since this procedure reduced BPS compared to higher temperatures. On the other hand, Silva et al. [72] showed that a combination of high vacuum pressure (9 mbar) and shrinking temperature (87 °C) can retard BPS.

## 9. Conclusions

Blown pack spoilage is still a big challenge for the meat industry. Future studies improving the culture conditions of *Cl. estertheticum* and exploring the genetic characteristics of *Cl. estertheticum* might provide further insights into the mechanisms that can be explored to control the growth *Cl. estertheticum* in vacuum-packs, and hence reduce the economic burden associated with BPS.

## Figures and Tables

**Table 1:** Composition of peptone yeast extract glucose starch.

Substance	g/L or mL/L
Proteose Peptone	5
Tryptone	5
Yeast extract	10
Meat extract Powder	10
Glucose	2
Soluble starch	1
Resazurin	0.001
Cysteine HCl	0.2
Solution of Silicon Antifoaming Agent 20%	0.25
Salts Solution A	40
Salts Solution B	40
Salts Solution A	
CaCl_2_·2H_2_O	0.265
MgSO_4_·7H_2_O	0.48
NaCl	2
Salts Solution B	
KH_2_PO_4_	1
K_2_HPO_4_·3H_2_O	1.3
NaHCO_3_	10

**Table 2:** Primers and probes used to detect *Cl. estertheticum*.

Assay	Primer and Probe		Sequence	Reference
PCR	Primer	16SEF	5′-TCG GAA TTT CAC TTT GAG-3′	[57]
		16SER	5′-AAG GAC TTC ACT CAT CTC TG-3′	
RT-PCR	Primer	TMF	5′-CGG CGG ACG GGT GAG TAA C-3′	[28]
		TMR	5′-CGG GTC CAT CTC AAA GTG RAA CT-3′	
	Probe		5′-FAM-CGT GGG TAA CCT GCC TCA AAG AGG GG-TAMRA-3′	
qPCR	Primer	TMF	5′-CGGCGGACGGGTGAGTAAC-3′	[25]
		Cl642-R	5′-CCTCTCCTGCACTCTAGA-3′	
	Probe	Cest	5′-HEX-CAAAGGAATTTTTCGGAATTTCACTTTGAG-BHQ1-3′	

PCR: Polymerase chain reaction; RT-PCR: Real time PCR; and qPCR; Quantitative PCR.
